# Identifying gaps and providing recommendations to address shortcomings in the investigation of acne sequelae by the Personalising Acne: Consensus of Experts panel

**DOI:** 10.1016/j.jdin.2021.06.006

**Published:** 2021-08-17

**Authors:** Alison Layton, Andrew Alexis, Hilary Baldwin, Stefan Beissert, Vincenzo Bettoli, James Del Rosso, Brigitte Dréno, Linda Stein Gold, Julie Harper, Charles Lynde, Diane Thiboutot, Jonathan Weiss, Jerry Tan

**Affiliations:** a Hull York Medical School, University of York, York, United Kingdom; b Harrogate and District NHS Foundation Trust, Harrogate, United Kingdom; c Weill Cornell Medicine, New York, New York; d Robert Wood Johnson Medical Center, New Brunswick, New Jersey; e The Acne Treatment and Research Center, Brooklyn, New York; f Department of Dermatology, University Hospital Carl Gustav Carus, Dresden, Germany; g Dermatology Unit – Teaching Hospital, Azienda Ospedaliera, University of Ferrara, Ferrara, Italy; h Thomas Dermatology, Las Vegas, Nevada; i JDR Dermatology Research, Las Vegas, Nevada; j Dermato-cancérology Department, CHU Nantes, University of Nantes, Nantes, France; k Henry Ford Health System, Detroit, Michigan; l Dermatology and Skin Care Center of Birmingham, Birmingham, Alabama; m Department of Medicine, University of Toronto, Ontario, Canada; n Lynderm Research Inc, Ontario, Canada; o Department of Dermatology, Pennsylvania State University College of Medicine, Hershey, Philadelphia; p Georgia Dermatology Partners, Snellville, Georgia; q Windsor Clinical Research Inc, Ontario, Canada; r Department of Medicine, University of Western Ontario, Ontario, Canada

**Keywords:** acne scarring, acne sequelae, acne-induced hyperpigmentation, acne-induced macular erythema, consensus, Delphi process, postinflammatory erythema, postinflammatory hyperpigmentation, PACE, Personalising Acne: Consensus of Experts

## Abstract

**Background:**

The physical sequelae of acne include erythema, hyperpigmentation, and scarring, which are highly burdensome for patients. Early, effective treatment can potentially limit and prevent sequelae development, but there is a need for guidance for and evidence of prevention-oriented management to improve patient outcomes.

**Objective:**

To identify unmet needs of acne sequelae and generate expert recommendations to address gaps in clinical guidance.

**Methods:**

The Personalizing Acne: Consensus of Experts panel of 13 dermatologists used a modified Delphi approach to achieve a consensus on the clinical aspects of acne sequelae. A consensus was defined as ≥75% of the dermatologists voting “agree” or “strongly agree.” All voting was electronic and blinded.

**Results:**

The panel identified gaps in current guidance and made recommendations related to acne sequelae. These included identification and classification of sequelae, pertinent points to consider for patient consultations, and management aimed at reducing the development of sequelae.

**Limitations:**

The recommendations are based on expert opinion and made in the absence of high-quality evidence.

**Conclusions:**

The identified gaps should help inform future research and guideline development for acne sequelae. The consensus-based recommendations should also support the process of consultations throughout the patient journey, helping to reduce the development and burden of acne sequelae through improved risk factor recognition, early discussion, and appropriate management.

**Capsule Summary**

• Acne sequelae are common and cause substantial burden to patients. However, evidence and guidance on prevention and management are sparse, potentially compromising patient outcomes.
• Clinical management gaps relating to acne sequelae were identified. Recommendations to enhance patient outcomes were made to improve sequelae classification, risk factor identification, and patient discussion.

## Introduction

Acne is a prevalent, chronic inflammatory skin condition that can lead to clinically relevant sequelae, such as erythema, hyperpigmentation, and scarring.^1^ Acne-induced scarring is most commonly atrophic but can also be hypertrophic or keloidal.^2^^,^^3^ Acne-induced macular hyperpigmentation, also termed “post-inflammatory hyperpigmentation,” describes an acquired hypermelanosis frequently attributed to prior cutaneous inflammation.^4^ Acne-induced macular erythema, also termed “post-inflammatory erythema,” represents persistent erythema as an initial acne lesion resolves.^5^

There have been few formal epidemiologic studies of acne sequelae; scarring is the most frequently investigated. The prevalence estimates for acne-induced scarring vary considerably, from 43% to 90.8%.^6^^,^^7^ Although scarring risk may correlate with increased acne severity, it also commonly occurs with mild or moderate disease.^6^^,^^7^ A number of other risk factors for acne scarring have also been identified, including time from acne onset and first effective treatment, disease relapse, family history, intensity of immune response mounted, lesion manipulation, and male sex.^6^^,^^8^^,^^9^ For acne-induced hyperpigmentation, patients with darker Fitzpatrick skin phototypes are more commonly affected than those with lighter Fitzpatrick skin phototypes,^10^, ^11^, ^12^, ^13^ whereas acne-induced erythema is generally considered to affect, or at least be more noticeable in, individuals with lighter Fitzpatrick skin phototypes.^5^^,^^12^ Patients frequently experience a combination of acne-induced sequelae.^5^^,^^12^

The burden of acne sequelae can be substantial.^1^ Because there can be a substantial discordance between a patient's and a physician's severity perceptions, objective assessments performed by a clinician may not be sufficient to assess individual burden.^14^ Acne-induced scars are perceived negatively in some societies, with affected individuals seen as less attractive, confident, happy, healthy, and successful than those without scars.^15^ Acne-induced hyperpigmentation is frequently long lasting and may prove to be more bothersome to the patient than the initial acne lesions.^1^^,^^12^^,^^13^ Similarly, acne-induced macular erythema is frequently considered cosmetically unacceptable and can contribute to the psychosocial burden of acne as well as have a significant and lasting impact on patients financially.^1^^,^^7^^,^^16^, ^17^, ^18^, ^19^ The treatment of acne sequelae often involves costly procedural interventions, such as laser, radiofrequency microneedling, and chemical peeling; however, high-quality evidence of the efficacy of such treatments is sparse.^5^^,^^12^^,^^19^^,^^20^ Furthermore, such interventions are often considered cosmetic in nature, which limits their accessibility to patients.^21^^,^^22^

Clinical guidelines for acne have suggested that early and effective treatment of acne can limit physical and psychosocial sequelae.^23^ However, acne is a chronic disease,^11^ and evidence of and guidance for the practical implementation of a long-term, patient-centered management plan in patients with acne are sparse.^24^, ^25^, ^26^ Without the best practice guidance, patients may receive suboptimal treatment and limited risk factor mitigation, potentially leading to higher risk of acne sequelae and psychologic comorbidity.^23^^,^^27^

As part of a 2020 consensus project, the Personalising Acne: Consensus of Experts (PACE) panelists aimed to identify the unmet needs in recognizing and managing acne sequelae and used an expert consensus, combined with the best available evidence, to address gaps in clinical guidance.

## Methods

### Expert panel

The expert panel consisted of 13 dermatologists from Canada (n = 2), France (n = 1), Germany (n = 1), Italy (n = 1), the United Kingdom (n = 1), and the United States (n = 7). Two cochairpersons from the main panel oversaw the process and were involved in panel selection and Delphi design.

### Modified Delphi process

**Fig 1:**
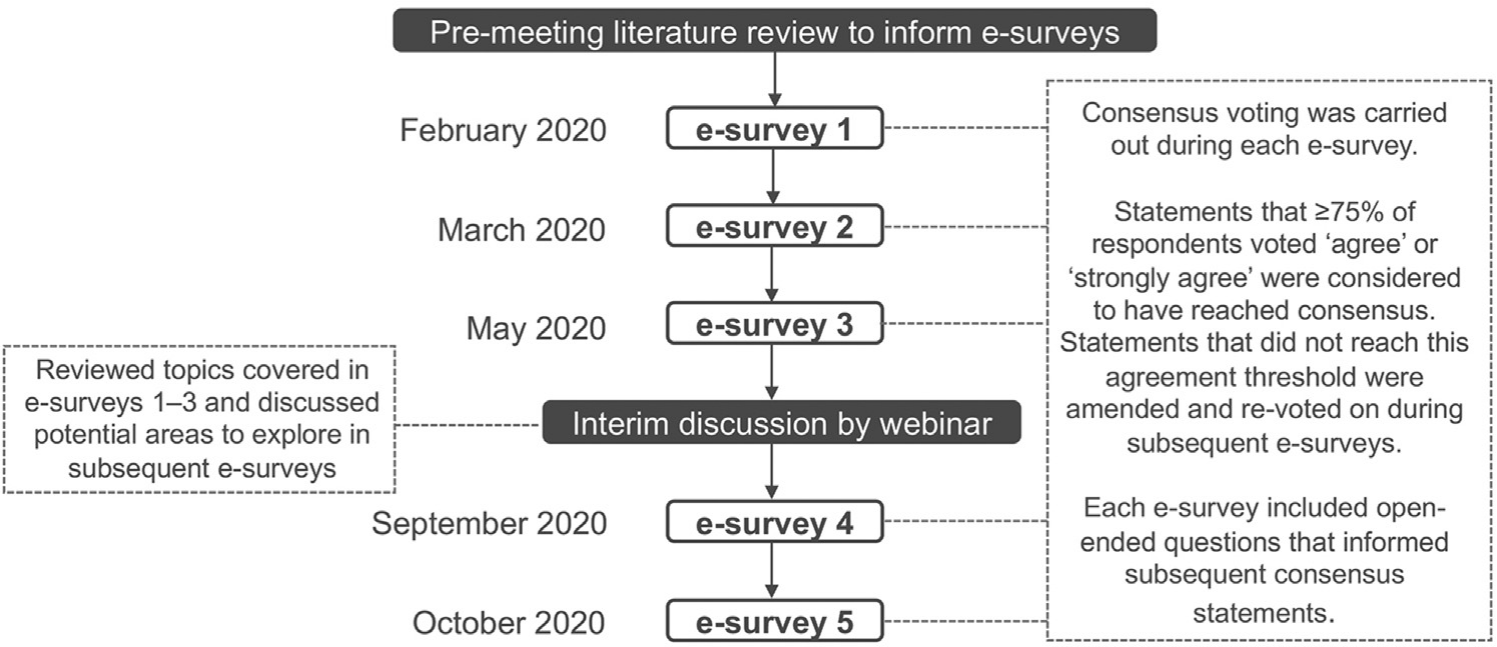
The Personalising Acne: Consensus of Experts modified Delphi process.

A modified Delphi process was used to reach a consensus on questions pertaining to acne sequelae identification, classification, and burden for patients as well as pertinent points to consider for patient consultation and management. The process consisted of a series of 5 e-surveys and an interim group webinar between the third and fourth e-survey (Fig 1). An initial literature search was conducted to identify clinical management gaps in acne and the need to make recommendations that incorporate all presentations of acne. The search process included an audit of acne clinical guidelines for Europe, the United States, and Canada^24^, ^25^, ^26^ to identify research gaps, followed by an additional assessment of relevant literature to address key clinical management questions associated with the gaps identified in the audit. The quality of evidence was rated according to the grading of recommendations assessment, development and evaluation (GRADE) methodology^28^ and used to guide the e-survey content. Further details on the process and outcomes of the literature search can be found in the Supplemental Information (available via Mendeley at https://data.mendeley.com/datasets/pg8658vmz9/2.)

### E-survey development and administration

Consensus statements were structured to assess the level of agreement using the following response range: “strongly disagree,” “disagree,” “agree,” “strongly agree,” or “unable to answer.” A consensus was defined as ≥75% of the panelists voting “agree” or “strongly agree.” Some questions were posed as multiple-choice questions, in which several responses could be selected; the results were presented as a consensus when chosen by ≥75% of the panelists. Some questions were open ended to allow for the development of consensus statements in a subsequent voting round. A virtual interim meeting was held after e-survey 3 to discuss the direction of the subsequent surveys. The e-surveys were programmed, administered, and the responses collated by Ogilvy Health UK in order to maintain blinding. The topic of acne sequelae was 1 of 4 major topics explored in the e-surveys and virtual interim meeting and will be the focus of this current article. Truncal acne, longitudinal management, and patient types were also covered and will be reported in subsequent publications.

## Results

### Definition of consensus recommendations

The result of the consensus statement voting is given in brackets (12/13 voted “agree” or “strongly agree”). Some panel members occasionally voted “unable to answer.” These votes were not included in the denominator. Full statements are available in the Supplemental Information (available via Mendeley at https://data.mendeley.com/datasets/pg8658vmz9/2.) The elements that were considered but not voted on are included in the “Discussion points” section below.

### Panelist demographics

Majority of the panelists reported that they had consulted publications for practical advice on managing acne sequelae and found them “somewhat helpful” (76.9%; 10/13). For the management of acne-induced scarring or acne-induced hyperpigmentation, 53.9% (7/13) did not find clinical practice guidelines useful; 38.5% (5/13) did not find them useful for the mitigation or prevention of future acne sequelae.

### Identification and classification of acne sequelae and their impact on patients

The gaps in and recommendations for identifying and classifying acne sequelae and their impact on patients are provided in Table I.

**Table I:** Gaps in and recommendations for identifying and classifying acne sequelae and their impact on patients, based on a consensus

Gaps • The term “postinflammatory” when describing hyperpigmentation in acne is a misnomer (11/12) • The term “postinflammatory” when describing erythema in acne is a misnomer (12/12) • Scarring is the single acne sequela that has the greatest impact on patients' quality of life (11/13) • If present, macular hyperpigmentation (10/12) in patients with darker skin phototypes (Fitzpatrick scale IV-VI), macular erythema (10/11), and scarring regardless of skin phototype (13/13) are some acne sequelae that have the greatest impact on patients' quality of life • The following are common (>50%) concerns reported by patients specifically with regard to acne sequelae: long-term or permanent duration (13/13); appearance (13/13); availability of options to treat them (11/13); and unmet expectations (eg, anticipated completely “perfect” skin; 10/13) Recommendations • The prefix “acne-induced” should be used to describe acne sequelae to differentiate the cause from other dermatologic conditions (13/13) • Acne-induced scars are volumetric changes (hypertrophic or atrophic) that occur on the skin as a result of primary acne lesions and may be permanent or may resolve over time or with treatment (12/13) • Residual dark marks or spots that occur on the skin as a result of acne lesions are more appropriately described as “macular hyperpigmentation” than “postinflammatory hyperpigmentation” (13/13) • Residual redness that occurs on the skin as a result of acne lesions is more appropriately described as “macular erythema” than “postinflammatory erythema” (12/12) • Macular erythema is a common sequela of acne (11/12) • Severe, inflammatory acne is a particular risk factor for acne-induced macular erythema (11/12) • Acne-induced macular erythema is typically more visible in patients with lighter skin phototypes (Fitzpatrick scale I-III) (11/12)

#### Discussion points

The panel recommended using the terminology “acne-induced macular erythema” and “acne-induced macular hyperpigmentation” in place of “post-inflammatory” to avoid conflating morphology with pathogenesis and to improve accuracy, since the term “macular” describes flat, circumscribed changes in skin color and inflammation occurring in patients with post-inflammatory erythema and post-inflammatory hyperpigmentation can be persistent as opposed to “post-inflammatory.” Furthermore, the prefix “acne-induced” provides more specificity.

Some panelists noted that acne-induced macular erythema might take a different hue in patients with darker skin, eg, purple or brown.

Patients seen by the PACE panelists typically express concerns about the permanence of acne-induced scars and other defects, such as discoloration. Resolution can be partial or complete, and sequelae may improve but not resolve, with or without treatment. Scarring generally has the longest duration of acne sequelae, although active inflammation and pigment changes notably impact the quality of life in the short term.

### Goals and patient consultations

The gaps in and recommendations for management goals and patient consultations concerning acne sequelae are provided in Table II.

**Table II:** Gaps in and recommendations for management goals and patient consultations concerning acne sequelae, based on a consensus

Gap • There is a need for better tools to support physicians in discussing the different types of acne sequelae with patients (13/13) Recommendations • Acne sequelae should be discussed with patients during the first consultation and revisited frequently (12/13) • The risk of acne-induced scarring (13/13), macular erythema (10/13), and macular hyperpigmentation (12/13) should be determined in all patients at diagnosis • Acne-induced macular hyperpigmentation is an important consideration when managing acne patients with darker skin phototypes (Fitzpatrick scale IV-VI) (13/13) • Where relevant and appropriate, the family history of acne should be discussed during consultations with patients with acne (13/13) • Reducing the risk of acne sequelae should be included as a goal for maintenance phase (12/13) • The following are essential elements to managing patients' expectations regarding acne sequelae: discuss their concerns about the effect of their disease (11/13); discuss their concerns around treatment (10/13); discuss their expectations of treatment (11/13); highlight that improvement may only be observed in the long term (10/13); be realistic with them about outcomes (11/13); emphasize the need for control of active acne to reduce the risk of developing sequelae (13/13); emphasize the role of modifiable risk factors (eg, lesion excoriation, adherence to medication) in reducing the risk of developing sequelae (13/13); and discuss management options for sequelae (10/13) • Visual aids (12/13) and digital aids (11/11) would be valuable tools to support discussions of different acne sequelae with patients • The following educational materials or tools would be most useful to support discussions of acne sequelae with patients: standardized definitions of sequelae (10/13); photographs (10/13); and apps (10/13)

#### Discussion points

The panelists typically discuss acne sequelae during the first consultation and in subsequent visits. However, during the first visit, it may be impractical or overwhelming to cover sequelae with certain patients. Therefore, some panelists may postpone discussion with certain individuals, such as younger patients or those with mild disease.

Early discussion of sequelae is important for a number of important reasons, including to help identify patients who are most at risk of developing sequelae using available clinical tools,^8^ set treatment expectations, provide advice for adhering to treatment (and the importance of adherence), avoid lesion manipulation, and highlight the need to gain control of active acne to minimize further sequelae development and reassure patients that they have some control in mitigating their risk of developing scars. Although many panelists considered it important to determine the risk of acne-induced macular erythema and hyperpigmentation in their patients at the time of diagnosis, scarring risk was considered the highest priority to discuss because scars are least likely to resolve over time without treatment.

### Treatment and management

The gaps in and recommendations for the treatment and management of acne sequelae are provided in Table III.

**Table III:** Gaps in and recommendations for the treatment and management of acne sequelae, based on a consensus

Gaps • There is a need for high-quality evidence for effective interventions to prevent (13/13) and manage existing (13/13) acne-induced scarring • There is a need for high-quality evidence for effective interventions to prevent (12/12) and manage existing (12/13) acne-induced macular hyperpigmentation • There is a need for high-quality evidence for effective interventions to prevent (11/12) and manage existing (10/11) acne-induced macular erythema Recommendations • Early intervention with effective treatments is an optimal approach for preventing acne sequelae (13/13) • A patient should be referred to a dermatologist when there is evidence of scarring (12/13)

#### Discussion points

Typically, panelists use early aggressive therapy with combination regimes targeting acne pathophysiology and adopt topical retinoids as part of the regimes to prevent acne scarring, moving to oral retinoids if the patient does not respond. The panelists did not vote for any statements relating to specific treatments for acne sequelae.

### General discussion

The sequelae of acne are common and can be long lasting and burdensome for patients. Here, the PACE panel identified gaps relating to acne sequelae recognition, classification, and discussion with patients and generated recommendations to improve patient care.

The key PACE recommendations are: those helping physicians identify patients with acne-induced scarring and scarring risk factors, eg, the presence of family history, and promoting the discussion of acne sequelae risk with patients early in the treatment journey. The discussion of sequelae with patients with acne can also help address patient-related factors that contribute to sequelae development, such as lesion manipulation and treatment nonadherence. The potential for acne sequelae requires greater forethought in patients with darker Fitzpatrick skin phototypes, which should form a part of their patient-centered management plan.^29^ The PACE panel recommended practical strategies to facilitate these discussions with patients and set realistic expectations for acne sequelae. In patients with existing or newly developed acne-induced scarring, the self-assessment of clinical acne-related scars and facial acne scar quality of life patient-oriented tools can help clinicians assess the severity and impact of acne scars^30^; however, tools for assessing other acne-induced sequelae are currently lacking.

Risk factors for acne sequelae have been identified^6^^,^^8^^,^^13^^,^^29^^,^^31^^,^^32^; the mitigation of their development depends on identifying patients who are most at risk as well as early and effective treatment of active acne. A risk assessment tool for acne scarring is under development by another group, which could help alert patients to the risk of scar development and has the potential to be a valuable public health measure, given the considerable associated morbidity.^33^

Acne-induced hyperpigmentation and acne-induced macular erythema have been identified as transitional lesions for atrophic scars.^31^ Although the pathogenesis of acne sequelae is complex, ongoing inflammation appears to be a key underlying cause of atrophic scar development.^9^ Indeed, even prior to acne lesion formation and in the absence of *Cutibacterium acnes*, inflammatory processes can be detected, and they persist through to scar formation.^34^ Similarly, multiple identified risk factors for acne-induced scarring, including longer duration to initiate effective treatment and disease relapse,^6^ indicate ongoing inflammation. Therefore, the PACE panel recommended that early treatment of active acne can help optimize patient outcomes, which is in line with the proposed pathophysiology of acne-induced sequelae development. Elsewhere, it has been proposed that treatment with topical agents, such as retinoids, benzoyl peroxide, and certain antibiotics, should form a part of the first-line treatment strategy to optimize outcomes for patients with acne.^35^ Now, there is evidence to suggest that retinoids play a role in acne atrophic scar repair in the absence of primary acne lesions^36^ and that fixed-dose combination, such as adapalene and benzoyl peroxide, have synergistic effects on acne lesions and the mitigation of acne scars.^35^

A strength of this project is the inclusion of experts from various countries who treat a range of patients across a spectrum of presentations in daily practice. Recommendations based on consolidated expertise using the egalitarian Delphi method can be considered an appropriate interim measure to support clinical management when high-quality evidence is either not available or not practical.^37^, ^38^, ^39^ However, some concerns have been expressed over bias and reproducibility in the Delphi process, stating that it is not necessarily an “evidence-based” process because it relies on clinical opinion.^40^^,^^41^ A potential limitation is that the panel did not fully represent a global perspective of acne (the experts being from Europe and North America); therefore, the recommendations and conclusions made may not be applicable on a global basis. A number of panelists frequently treat patients across the spectrum, including those with darker Fitzpatrick skin phototypes, which led to recommendations for recognizing and managing acne sequelae in patients with darker Fitzpatrick skin phototypes and is itself a major strength of the PACE recommendations. However, perspectives from experts practicing in regions beyond North America and Europe would have added valuable insights, not only for recognizing and managing acne sequelae in patients with other Fitzpatrick skin phototypes but also for clinical, cultural, and systemic health care system practices that can influence acne management. In addition, these recommendations did not include the patients' perspective, which can also add a valuable insight when incorporated.

The present panel also recommended several areas for further work specific to acne sequelae. Although recommendations for treatment options to prevent and manage acne sequelae are available in the literature,^42^^,^^43^ additional high-quality evidence is needed to support evidence-based clinical management guidelines. The recommendations herein could additionally be used to inform the development of future treatment algorithms for acne sequelae. Visual and digital aids were suggested as useful tools to support discussions of acne sequelae with patients. However, it is important to consider whether such tools may induce stress and anxiety in certain individuals, which might be mitigated through use of cartoonized versions. Furthermore, there may be a benefit in terms of aids that can be provided directly to patients, thus relieving some of the burden on the physician. The distribution of the updated sequelae lexicon can help standardize terminology among physicians and support conversations with patients by enhancing their understanding of sequelae. Acronyms of the new terminologies may be useful in clinical practice. Such acronyms should be intuitive and differentiated from existing medical acronyms (eg, AISc [acne-induced scarring], AIMacPig [acne-induced macular pigmentation], and AIMacEry [acne-induced macular erythema]). Of note, an individual care pathway is currently under development by the PACE panel, which will include acne sequelae as a consideration for holistic patient management throughout the treatment journey.

## Conclusions

The PACE panel identified gaps that can help guide further work and research in the field of acne sequelae identification, prevention, and management. These recommendations can support local guideline development and patient consultations, thus helping to relieve the burden of acne sequelae in patients through improved risk factor recognition, early discussion, and prevention at all stages of the patient's journey.

## Conflict of interest

All panel members received honoraria from Galderma for participating in this project. Dr Layton has acted as an advisor or consultant, been a chief investigator for research (funded to institution), and/or received honoraria for unrestricted educational events from 10.13039/501100009754Galderma, La Roche-Posay, L'Oreal, LEO Pharma, Cipher, Proctor and Gamble, Almirall, GSK, and Origimm. Dr Alexis has received grant/research support from LEO Pharma, Novartis, Almirall, Bristol-Myers Squibb, Amgen, Menlo, 10.13039/501100009754Galderma, Valeant (Bausch Health), Cara and Arcutis; has acted as a consultant for LEO Pharma, Novartis, Menlo, Galderma, Pfizer, Sanofi-Regeneron, Dermavant, Unilever, Beiersdorf, Valeant, L'Oreal, Bristol-Myers-Squibb, Menlo, Scientis, Bausch health, UCB, Foamix, Cassiopea, Arcutis, Janssen, Allergan, Almirall, AbbVie and Sol-Gel; and has acted as a speaker (unbranded) for Regeneron, SANOFI-Genzyme, Pfizer, and AstraZeneca. Dr Baldwin has acted as an investigator, consultant, and/or speaker for Almirall, Bausch, Cassiopea, EPI Health, Galderma, La Roche-Posay, L'Oreal, Mayne Pharma, Sol-Gel, Sun Pharma, and Vyne. Dr Beissert has acted as an advisory board member for AbbVie Deutschland GmbH and Co KG, Actelion Pharmaceuticals Deutschland GmbH, Amgen GmbH, Celgene GmbH, Galderma Laboratorium GmbH, Janssen-Cilag GmbH, LEO Pharma GmbH, Lilly Deutschland GmbH, Novartis Pharma GmbH, MSD Sharp and Dohme GmbH, Menlo Therapeutics, Sanofi-Aventis Deutschland GmbH, Pfizer Pharma GmbH, and UCB Pharma GmbH and has received speaker honorarium from Novartis Pharma GmbH, AbbVie Deutschland GmbH and Co KG, MSD Sharp and Dohme GmbH, Pfizer Pharma GmbH, Janssen-Cilag GmbH, Galderma Laboratorium GmbH, Celgene GmbH, La Roche-Posay Laboratoire Pharmaceutique, Actelion Pharmaceuticals Deutschland GmbH, GlaxoSmithKline GmbH and Co KG, Bristol-Myers Squibb GmbH and Co KGaA, Sanofi-Aventis Deutschland GmbH, Almirall-Hermal GmbH, and Sandoz/HEXAL AG. Dr Bettoli has acted as a consultant, advisory board member, and research investigator; received honoraria from AbbVie, Baiersdorf, Bioderma, Biogena, Difa-Cooper, Galderma, GSK, ICF, LEO Pharma, L'Oreal, Meda, Menarini – Relife, Mylan, Novartis, Pharcos-Biodue, UCB Pharma; and received research support (funds to institution) from AbbVie. Dr Rosso has acted as a research investigator, consultant, and/or speaker for Almirall, Bausch Health (Ortho Dermatology), BiopharmX, EPI Health, Galderma, LEO Pharma, Mayne Pharma, Sol-Gel, Sonoma, Sun Pharma, and Vyne Therapeutics (Foamix). Dr Dréno has acted as a consultant for Galderma. Dr Gold has acted as an investigator or advisor and/or speaker for Galderma, Ortho Derm, Sun Pharma, Sol-Gel, Foamix, Novartis, and Almirall. Dr Harper has acted as a consultant for Almirall, BioPharmX, Cassiopea, Cutera, EPI, Foamix, Galderma, Ortho, Sol-Gel, and Sun Pharma. Dr Lynde has acted as a principal investigator, speaker, and consultant for Cipher Pharma, Bausch Health, Galderma, Johnson and Johnson, GSK, and Valeant. Dr Thiboutot has acted as a consultant for Cassiopea, Galderma, and Novartis. Dr Weiss has acted as an investigator or advisor and/or speaker for Galderma, Ortho Derm, Foamix, Novartis, Almirall, Dr. Reddy's, and EPI Health. Dr Tan has acted as an advisor, consultant, investigator, and/or speaker and received grants/honoraria from Bausch, 10.13039/501100009754Galderma, Pfizer, Almirall, Boots/Walgreens, Botanix, Cipher Pharmaceuticals, 10.13039/501100009754Galderma, Novan, 10.13039/100004336Novartis, Promius, Sun Pharma, and Vichy.
